# Effect of Phenylephrine and Ephedrine on Cerebral (Tissue) Oxygen Saturation During Carotid Endarterectomy (PEPPER): A Randomized Controlled Trial

**DOI:** 10.1007/s12028-019-00749-w

**Published:** 2019-06-12

**Authors:** Leonie M. M. Fassaert, Gert J. de Borst, Claire W. A. Pennekamp, Jantine C. Specken-Welleweerd, Frans L. Moll, Wilton A. van Klei, Rogier V. Immink

**Affiliations:** 1 Department of Vascular Surgery G04.129, University Medical Center Utrecht, University of Utrecht, Heidelberglaan 100, 3584 CX Utrecht, The Netherlands; 2 Department of Anesthesiology, University Medical Center Utrecht, Utrecht, The Netherlands; 3 Department of Medical Biology, Laboratory for Clinical Cardiovascular Physiology, Amsterdam University Medical Center, Amsterdam, The Netherlands; 4 Department of Anesthesiology, Amsterdam University Medical Center, Amsterdam, The Netherlands

**Keywords:** Carotid endarterectomy, Cerebral autoregulation, Vasopressors, Oxygen, Cerebral

## Abstract

**Background:**

Short-acting vasopressor agents like phenylephrine or ephedrine can be used during carotid endarterectomy (CEA) to achieve adequate blood pressure (BP) to prevent periprocedural stroke by preserving the cerebral perfusion. Previous studies in healthy subjects showed that these vasopressors also affected the frontal lobe cerebral tissue oxygenation (rSO_2_) with a decrease after administration of phenylephrine. This decrease is unwarranted in patients with jeopardized cerebral perfusion, like CEA patients. The study aimed to evaluate the impact of both phenylephrine and ephedrine on the rSO_2_ during CEA.

**Methods:**

In this double-blinded randomized controlled trial, 29 patients with symptomatic carotid artery stenosis underwent CEA under volatile general anesthesia in a tertiary referral medical center. Patients were preoperative allocated randomly (1:1) for receiving either phenylephrine (50 µg; *n *= 14) or ephedrine (5 mg; *n *= 15) in case intraoperative hypotension occurred, defined as a decreased mean arterial pressure (MAP) ≥ 20% compared to (awake) baseline. Intraoperative MAP was measured by an intra-arterial cannula placed in the radial artery. After administration, the MAP, cardiac output (CO), heart rate (HR), stroke volume, and rSO_2_ both ipsilateral and contralateral were measured. The timeframe for data analysis was 120 s before, until 600 s after administration.

**Results:**

Both phenylephrine (70 ± 9 to 101 ± 22 mmHg; *p *< 0.001; mean ± SD) and ephedrine (75 ± 11 mmHg to 122 ± 22 mmHg; *p *< 0.001) adequately restored MAP. After administration, HR did not change significantly over time, and CO increased 19% for both phenylephrine and ephedrine. rSO_2_ ipsilateral and contralateral did not change significantly after administration at 300 and 600 s for either phenylephrine or ephedrine (phenylephrine 73%, 73%, 73% and 73%, 73%, 74%; ephedrine 72%, 73%, 73% and 75%, 74%, 74%).

**Conclusions:**

Within this randomized prospective study, MAP correction by either phenylephrine or ephedrine showed to be equally effective in maintaining rSO_2_ in patients who underwent CEA.

*Clinical Trial Registration* ClincalTrials.gov, NCT01451294.

## Introduction

In most patients scheduled for carotid endarterectomy (CEA), both baroreflex sensitivity (BRS) and cerebral autoregulation (CA) are impaired [1, 2]. This results in blood pressure (BP) fluctuations that cannot be counter-regulated by the brain vasculature [3]. Therefore, during a CEA procedure systemic hemodynamics should be optimized. A rule of thumb is to keep the mean arterial pressure (MAP) between preoperative awake values upwards to 20% above baseline [4, 5]. However, it can be challenging to achieve this targeted BP level intraoperatively due to induction medication and anesthetics [1]. Thus, short-acting vasopressors like phenylephrine or a combined vasopressor and positive inotropic agent like ephedrine are administered in relatively large quantities. Despite that both vasopressor agents effectively elevate MAP, there is mounting evidence that frontal lobe cerebral tissue oxygenation (rSO_2_), measured by near-infrared spectroscopy (NIRS), decreases during the administration of phenylephrine while it remains unaffected during ephedrine use [6, 7].

The mechanism behind this observation remains unclear. In patients with intact CA, the decrease in rSO_2_ after phenylephrine was associated with concordant changes in cardiac output (CO), whereas rSO_2_ remained unchanged when CO remained constant after treatment with ephedrine [6]. This observation confirms that changes in CO, even independently from arterial pressure, affect cerebral hemodynamics [8, 9]. In both healthy and acute stroke patients, CO seemed to contribute to the regulation of the cerebral blood flow. Also, cerebral arteries are abundantly innervated by sympathetic fibers [10]. The decrease in rSO_2_ after phenylephrine could be explained by a direct α_1_-receptor-mediated cerebral vasoconstriction. In this respect, this would confirm a possible blood–brain barrier permeability for α_1_-receptors–agonists and the presence of α-receptors in the smooth muscle layer of the cerebral vessels and microcirculation [11, 12].

In a small exploratory case series, addressing the cerebral hemodynamics of both vasopressors agents during CEA, a detrimental effect of phenylephrine consisting of a decrease in rSO_2_ after administration in CEA patients has been described [13]. Therefore, the present blinded randomized controlled study aimed to evaluate the previous observation of rSO_2_ remaining unaffected after ephedrine and declining after phenylephrine when administrated for the treatment of a hypotensive period perioperative in patients undergoing CEA.

## Materials and Methods

### Subjects

Ethical approval for this study (NL37658.041.11) was provided by the Medical Research Ethics Committee of University Medical Center Utrecht, Utrecht, The Netherlands (Chairperson Dr. W. A. Groenewegen), on July 30 2012. Following approval, informed consent was obtained from 42 patients undergoing CEA between October 2012 and September 2013 at a tertiary referral vascular center, the University Medical Center Utrecht. The protocol of our randomized study was registered (Clinicaltrials.gov:NCT01451294) and has been published previously [14]. In short, patients with asymptomatic stenosis (> 70%) or symptomatic stenosis (> 50%) of the carotid artery scheduled for CEA were eligible for inclusion. Indications for carotid revascularization were discussed in a multidisciplinary team consisting of neurologists, radiologists, and vascular surgeons. Exclusion criteria were: intraoperative decrease in MAP (expressed in mmHg) of less than 20% compared to baseline, arrhythmia or hypersensitivity to either ephedrine or phenylephrine [14].

### Carotid Endarterectomy

All patients were operated under volatile general anesthesia (GA) and received standard monitoring (noninvasive arterial BP with an upper arm cuff, electrocardiogram, pulse oximetry, end-tidal carbon dioxide, and temperature). Prior to anesthetic induction, an intra-arterial cannula (20 G) was placed in the radial artery to monitor invasive continuous beat-to-beat blood pressure (ABP). Electroencephalography (EEG, Micromed Inc., Treviso, Italy) electrodes continuously registered during surgery to monitor cerebral function state and detecting signs of cerebral ischemia. Detailed information on volatile GA and the surgical procedure is described previously in the protocol [14]. Intraoperatively, all patients received intravenous low-dose norepinephrine as part of standard care.

### Study Design

**Fig. 1:**
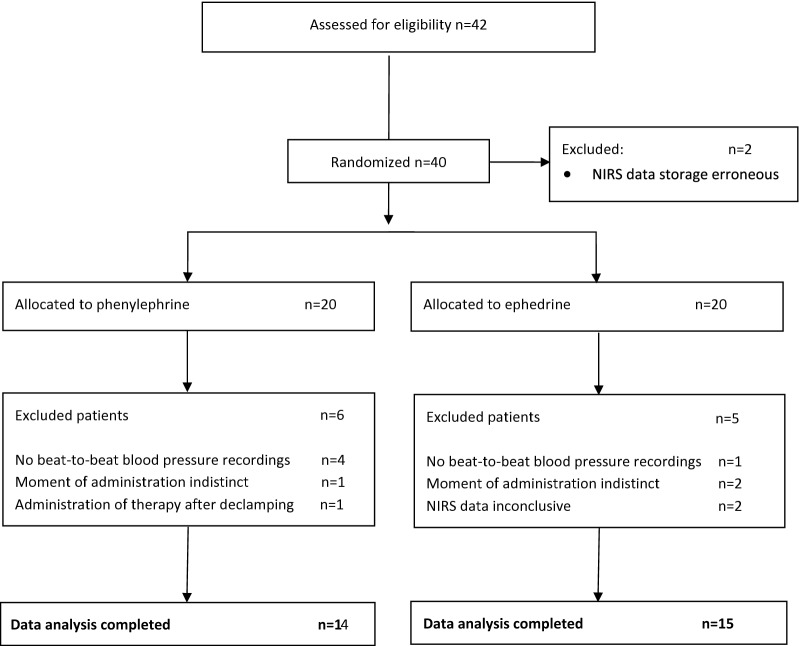
Flowchart of study

The study aimed to investigate the impact of ephedrine and phenylephrine on rSO_2_ when administrated for correction of intraoperative hypotension in patients undergoing CEA. Secondary outcome measures of neurological or hemodynamic compromise in the postoperative phase were not analyzed in this study. Patients were allocated randomly (1:1) by computer-generated randomization for receiving either phenylephrine or ephedrine when intraoperative hypotension occurred before carotid cross-clamping. Relative intraoperative hypotension requiring intervention was defined as a decrease in MAP of ≥ 20% compared to awake baseline MAP (Fig. 1). The baseline MAP was the measured noninvasive BP on the upper arm, ipsilateral to the operation side, on the ward 1 day before surgery. When intraoperative hypotension occurred despite low-dose intravenous support of norepinephrine (hypotensive episode had to occur before cross-clamping when attending in the study), the attending anesthesiologist, blinded for the study medication, administered 1 ml from a prepared 10-ml unlabeled syringe containing either phenylephrine (50 µg·ml^−1^) or ephedrine (5 mg·ml^−1^). This syringe was prepared by a co-worker not involved in the study. The chosen dose of both vasopressors was based on the relative potency ratio for phenylephrine:ephedrine of 80:1 [15]. The chosen timeframe for data analysis was 120 s before, until 600 s after administration.

If a patient did not respond within 5 min after the first bolus, a second bolus of the same vasopressor was given. If so, the timeframe around the second bolus was used for the data analysis. When hypotension persisted, the patient was classified as non-responder and rescue medication, as preferred by the attending anesthesiologist, was administered. Non-responders were not used for data analysis.

### Intraoperative Measurements

Details on intraoperative monitoring are as described in the protocol [14] and follows:

#### Hemodynamic

The radial ABP curve, sampled with 100 Hz, was derived from the Data Ohmeda S/5™ monitoring system (GE Healthcare, Waukesha, Wisconsin, USA) and stored for offline analysis. MAP was measured as the mean integral over one heartbeat. Using the model flow method CO, stroke volume (SV), heart rate (HR), and systemic vascular resistance (SVR) were determined by BeatFast (TNO TPD Biomedical Instrumentation, Amsterdam, The Netherlands). HR was determined as the inverse of the inter-beat-interval and expressed in beats per minute (bpm). SV was calculated from the ABP waveform incorporating age, sex, height, and weight of the patients [16, 17]. The CO was calculated as the product of SV and HR. The SVR was the ratio of MAP to CO.

#### Near-Infrared Spectroscopy

Two NIRS optodes (Invos 3100; Somanetics Corporation, Troy, MI, USA) were placed bilaterally on the forehead to measure rSO_2_ previous to induction. These optodes allowed continued monitoring of the rSO_2_ by emitting two wavelengths of near-infrared light (730 and 805 nm) from two separate (3 and 4 cm) diode sources to a receiver [18]. NIRS output was sampled at 0.16 Hz.

### Data Analysis

The offline radial ABP curve of each patient was synchronized with the rSO_2_ signal, using time markers, which were applied intraoperatively. Moment of administration was marked. The timeframes (− 120 to 600 s) for data analysis of both curves were retrieved. The beat-to-beat data were averaged over 360 slots of 2 s. By polynomial interpolation, the rSO_2_ signals were divided into 72 slots of 10 s.

### Statistical Analysis

Sample size calculation was based on a retrospective pilot study [13]. This retrospective pilot study showed a decrease in rSO_2_ of − 1.5% (± 2) per 10 mmHg increase after administration of phenylephrine [14]. Based on this calculation, 14 patients in each group were needed to detect a significant decrease in rSO_2_ after administration of phenylephrine (α level 0.05 and probability power 0.9). Patients who did not receive vasopressor agents intraoperative or failure of rSO_2_ measurements occurred during surgery for reasons unrelated to the surgical procedure were replaced according to protocol [14].

All analyses are performed according to the intention-to-treat principle. Results are mean ± SD for normally distributed data and median (range) for data not normally distributed. Changes in CO, SV, and SVR are presented as percentage change from baseline. Delta (Δ) of rSO_2_, MAP, HR, and CO were calculated at different time points, namely the moment of maximum increase in BP, 5 min and 10 min after administration. Wilcoxon signed-rank test determined multiple pairwise comparisons. Student’s paired *t* test was used to evaluate changes between conditions, and a confidence level of less than 5% (0.05) was considered significant. To compare ΔrSO_2_ between phenylephrine and ephedrine, Student’s *t* test is used for normal distributed data, and Wilcoxon signed-rank test or Mann–Whitney *U *for non-normal distributed data (paired/non-paired). Use of pacemaker and beta-blockers was taken into account and described separately. The statistical analysis was performed using Statistical Package for Social Sciences version 22.0 (SPSS Inc. Chicago, IL, USA).

## Results

### Patient Characteristics

Written informed consent was obtained from 42 patients. NIRS technical failure occurred before randomization in two cases, and 11 patients were excluded after randomization (Fig. 1). Baseline characteristics of the excluded patients did not significantly differ from the included patients. A total of 29 patients (19 male) with symptomatic carotid artery stenosis were enrolled for the final data analysis (Table 1). Except for history of peripheral vascular disease (PVD), the two groups were similar to each other in perspective of baseline characteristics. Preoperative MAP was higher in the ephedrine group. In accordance with the protocol [14], all patients had a decrease in MAP of ≥ 20%. Ephedrine was administered to 15 patients, and 14 patients received phenylephrine.

**Table 1:** Baseline characteristics

	Ephedrine (*n *= 15)	Phenylephrine (*n *= 14)
Age (years)	72 ± 8	71 ± 9
Gender (male)	7 (47%)	12 (86%)
BMI (kg m^−2^)	27 ± 5	27 ± 4
DMII	4 (27%)	7 (50%)
Hypertension	10 (67%)	11 (79%)
Hypercholesterolemia	3 (20%)	8 (57%)
CAD	2 (13%)	5 (36%)
PVD	–	4 (29%)
AF	2 (13%)	1 (7%)
Pacemaker	2 (13%)	–
Smoking		
Current	6 (40%)	8 (57%)
Past	5 (33%)	6 (43%)
Alcohol	6 (40%)	9 (64%)
Preoperative ß-blocker	3 (20%)	6 (43%)
Statin use	11 (73%)	13 (93%)
Operation side (right)	5 (33%)	6 (43%)
Symptomatic (yes)	15 (100%)	14 (100%)
Degree of ipsilateral stenosis		
> 95%	2 (13%)	5 (36%)
70–95%	13 (87%)	7 (50%)
50–70%	–	2 (14%)
Degree of contralateral stenosis		
Occlusion	–	–
70–99%	–	1 (7%)
< 70% or N.A.	15 (100%)	13 (93%)
Shunt use	2 (13%)	2 (14%)
NE infusion (µg kg^−1^ min^−1^)	0.05 ± 0.03	0.05 ± 0.03
Isoflurane *n* (%)	6 (40%)	5 (36%)
Isoflurane dose (%)	0.61% (IQR 0.20)	0.79% (IQR 0.53)
Isoflurane MAC value	0.51 (IQR 0.17)	0.68 (IQR 0.45)
Sevoflurane *n* (%)	9 (60%)	9 (64%)
Sevoflurane dose (%)	1.65% (IQR 0.62)	1.46% (IQR 0.75)
Sevoflurane MAC value	0.66 (IQR 0.25)	0.58 (IQR 0.30)
Non-responders	–	–
Preoperative systole (mmHg)	154 ± 13	142 ± 19
Preoperative diastole (mmHg)	83 ± 14	75 ± 12
Preoperative MAP (mmHg)	107 ± 12	97 ± 12

Values are shown as mean (± SD) or number of patients (%)

*AF* atrial fibrillation, *BMI* body mass index, *CAD* coronary artery disease, *DMII* diabetes mellitus type II, *IQR* inter quartile range, *MAC value* minimum alveolar concentration, *MAP* mean arterial pressure, *NE infusion* intravenous norepinephrine infusion rate at moment of administration of the study medication, *PVD* peripheral vascular disease

### Ephedrine

**Fig. 2:**
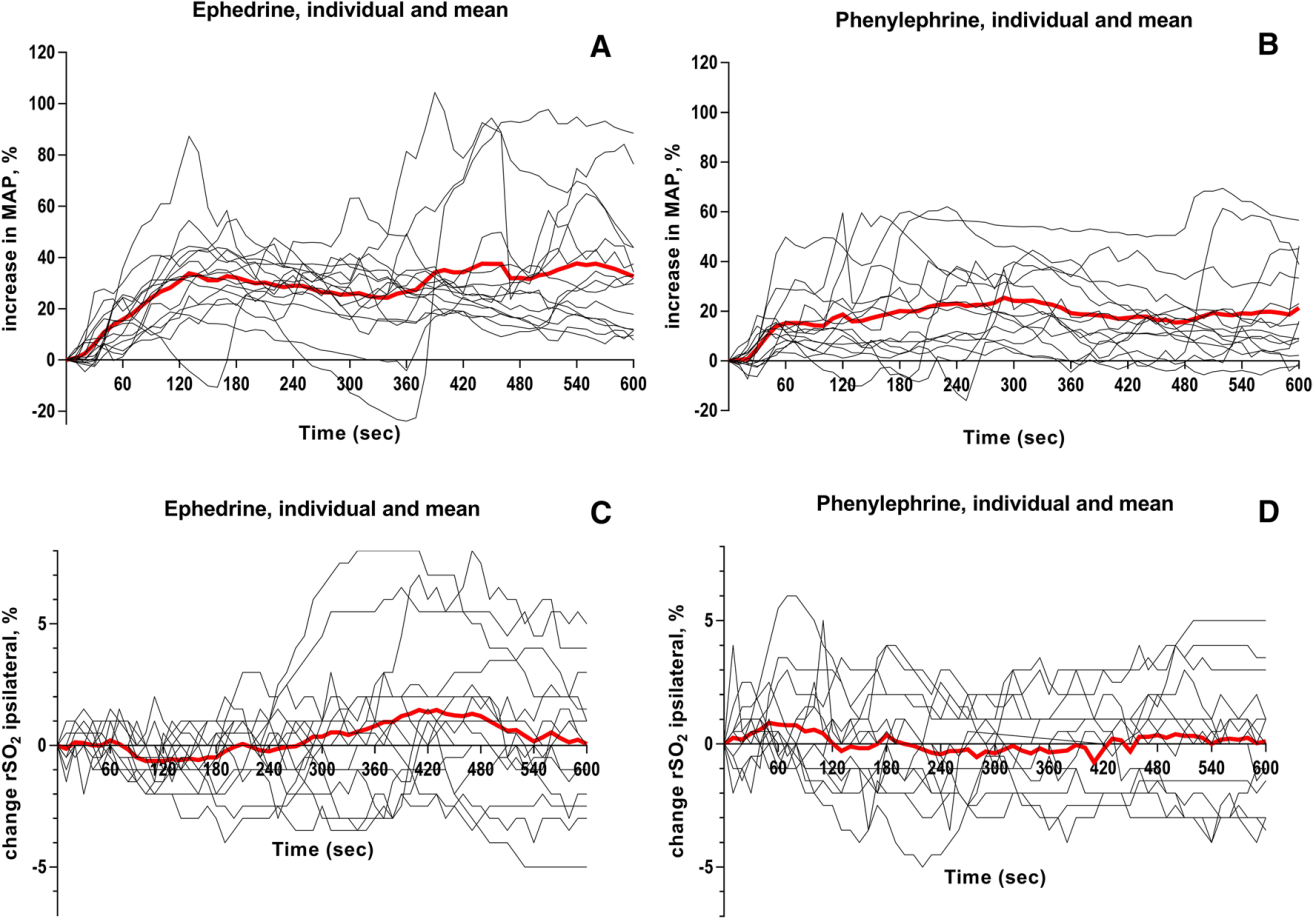
Percentile changes in mean arterial blood pressure (MAP) (**A**–**B**) and rSO_2_ ipsilateral (%) (**C**–**D**) individually and mean for both ephedrine and phenylephrine over time (s). Data in mean.

**Fig. 3:**
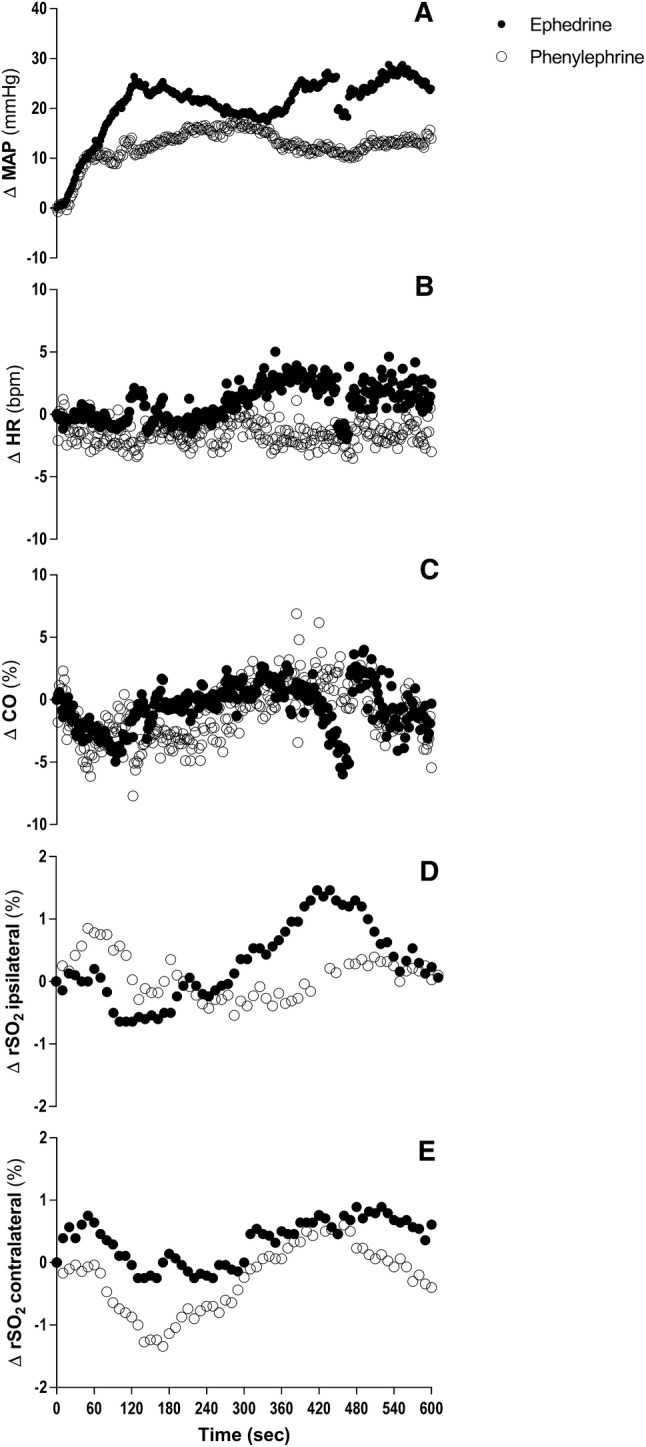
Changes in mean arterial blood pressure (MAP) (**A**), heart rate (HR) (**B**), percentile change in cardiac output (CO) (**C**), and frontal cerebral lobe oxygenation ipsilateral (**D**) and contralateral (**E**) during intravenously administration of ephedrine (filled circles) and phenylephrine (open circles) over time. Data in mean.

Awake MAP in the patients receiving ephedrine was 107 ± 12 mmHg. A single bolus was administered in 13 subjects and a second bolus in 2 subjects. This led to restoration of MAP to 122 ± 22 mmHg. Both ipsilateral rSO_2_ and contralateral rSO_2_ did not change significantly over time, showing a maximum decrease after administration of − 2.3% and − 2.5% and a maximum increase of 3.1% and 2.5%, respectively. The highest CO monitored after ephedrine administration was 118 ± 10% compared to baseline (*p *< 0.01). This rise in CO did not influence ipsilateral rSO_2_ (*p *= 0.107). No non-responders were reported (Table 2, Figs. 2, 3).

**Table 2:** Systemic and cerebral hemodynamics

	Baseline	Drug administration	300 s post	600 s post	Highest MAP	Highest CO	Lowest rSO_2_
Ephedrine (*n *= 15)							
MAP (mmHg)	80 ± 14	75 ± 11	94 ± 17*	99 ± 23*	122 ± 22*	95 ± 21	98 ± 21
CO (%)	100 ± 5	99 ± 4	99 ± 14	98 ± 10	93 ± 19	118 ± 10^†^	95 ± 11
HR (min^−1^)	61 ± 8	61 ± 8	62 ± 10	63 ± 9	68 ± 11*	69 ± 10*	60 ± 9
SV (%)	101 ± 3	100 ± 4	98 ± 9	96 ± 10	85 ± 17*	102 ± 12	97 ± 9
SVR (%)	101 ± 8	96 ± 5	122 ± 24^†^	128 ± 32^†^	177 ± 85*	103 ± 9	132 ± 38
rSO_2_ (%) ipsi	73 ± 9	72 ± 9	73 ± 8	73 ± 7	73 ± 8	74 ± 8	70 ± 8*
rSO_2_ (%) contra	75 ± 11	75 ± 10	74 ± 10	74 ± 10	74 ± 9	75 ± 10	71 ± 10*
Phenylephrine (*n *= 14)							
MAP (mmHg)	76 ± 13	70 ± 9	87 ± 13*	84 ± 14*	101 ± 22*	83 ± 13	76 ± 13
CO (%)	101 ± 4	98 ± 6	99 ± 9	93 ± 11	96 ± 9	117 ± 19	95 ± 14
HR (min^−1^)	57 ± 11	58 ± 14	58 ± 14	55 ± 9	59 ± 13	64 ± 17^†^	56 ± 13
SV (%)	101 ± 5	100 ± 7	99 ± 10	96 ± 9	92 ± 10^†^	102 ± 11	95 ± 14
SVR (%)	104 ± 7	96 ± 10	123 ± 19*	125 ± 27^†^	140 ± 24*	101 ± 18	113 ± 22
rSO_2_ (%) ipsi	74 ± 7	73 ± 7	73 ± 8	73 ± 8	73 ± 7	74 ± 7	71 ± 7*
rSO_2_ (%) contra	74 ± 8	73 ± 8	73 ± 8	74 ± 8	72 ± 9	74 ± 9	72 ± 8^†^

Systematic and cerebral hemodynamic variables in 120 s before administration (baseline), on the moment of administration (drug injection), at 300 s and 600 s after administration, during the highest mean arterial pressure (MAP), during the highest cardiac output (CO), and during the lowest frontal cerebral lobe oxygenation (rSO_2_). Data presented as mean ± SD

*CO* cardiac output, *HR* heart rate, *MAP* mean arterial pressure, *rSO*_*2*_ cerebral tissue perfusion, *SV* stroke volume, *SVR* systemic vascular resistance

*p value < 0.001; ^†^*p* value < 0.05 compared to administration

Two patients had a pacemaker that was switched to a fixed rate of, respectively, 60 and 70 min^−1^. Three patients used β-blockers. At the moment of administration, there was no difference (*p *= 0.77) in HR (59 min^−1^ vs. 61 min^−1^) and rSO_2_ both ipsilaterally and contralaterally (68 ± 9% vs. 74 ± 8% ipsilateral, 69 ± 11% vs. 76 ± 10% contralateral) between patients with and without β-blockers. MAP was lower at the administration of ephedrine in patients without β-blockers (*p *= 0.311, 74 mmHg vs. 80 mmHg). In addition, the same applied for ipsilateral and contralateral rSO_2_ measurements over time and β-blocker use.

### Phenylephrine

Awake MAP in the patients receiving phenylephrine was 97 ± 12 mmHg. A single bolus of phenylephrine was administered in 12 subjects and a second bolus in 2 subjects. MAP at the moment of administration was 70 ± 9 mmHg. This led to restoration of MAP to 101 ± 22 mmHg (*p *= 0.01). The highest CO monitored after phenylephrine administration was 117 ± 19% (*p *= 0.01). This increase in CO resulted in an insignificant change in ipsilateral and contralateral measured rSO_2_ with a maximum decrease in ipsilateral rSO_2_ of − 2.3% and a maximum increase of 2.8% and contralateral rSO_2_ − 1.8% and 2.6%, respectively. HR changed significantly from 58 ± 14 to 64 ± 17 min^−1^ at the highest CO (*p *= 0.019). No non-responders were reported (Table 2 and Figs. 2, 3).

Six patients used β-blockers before surgery. On the moment of administration, HR was significantly lower in the patients with β-blockers versus patient without β-blockers, 49 versus 64 min^−1^ (*p *= 0.028). The highest CO measured after administration was 109 ± 8% for the patients using β-blockers and 125 ± 23% for patients not using β-blockers (*p *= 0.156). HR did not change significantly over time in patients with β-blockers and patients without β-blockers usage (β-blockers at 300 s, 600 s; *p *= 0.257 and *p *= 0.167, respectively, without β-blockers at 300 s, 600 s; *p *= 0.233 and *p *= 0.326, respectively). Of ipsilateral and contralateral rSO_2_ measured over time, no difference was observed for β-blockers use. Restoration MAP was lower in the patients using β-blockers (87 ± 12 mmHg vs. 111 ± 22 mmHg, *p *= 0.039). Four patients in the phenylephrine arm had a history PVD. In these patients, rSO_2_ ipsilateral was significantly lower at administration (67 ± 4% vs. 75 ± 7%), minimum (64 ± 4% vs. 73 ± 7%) and maximum (70 ± 5% vs. 78 ± 6%) measured rSO_2_ after administration compared to patients without PVD. However, the absolute change of ipsilateral measured rSO_2_ did not differ between groups over time. MAP and contralateral measured rSO_2_ did not differ for patients with or without PVD.

### Differences Between Ephedrine and Phenylephrine

Effects on systemic and cerebral hemodynamic parameters were compared between the two treatment arms. The maximum increase in MAP after administration was significantly higher in the ephedrine group (*p *= 0.016). Changes in HR and CO after administration did not differ significantly between groups. After adjustment of preoperative beta-blocker medication use, the results did not change and no significant interaction was found. Additionally, changes in rSO_2_ both ipsilateral and contralateral did not show any significant differences over time after administration between the ephedrine and phenylephrine groups (Table 3, 4, 5 and Figs. 2, 3).

**Table 3:** Cerebral oxygenation over time between ephedrine group and phenylephrine group

	Ephedrine (*n *= 15)	Phenylephrine (*n *= 14)	*p* value
Cerebral perfusion: ipsilateral to surgery			
rSO_2_ at baseline (%)	73 ± 9	74 ± 7	0.734
rSO_2_ at administration (%)	72 ± 9	73 ± 7	0.848
Lowest rSO_2_ (%)	70 ± 8	71 ± 7	0.839
Highest rSO_2_ (%)	76 ± 7	76 ± 7	0.912
Mean rSO_2_ after administration (%)	73 ± 7	73 ± 7	0.928
Restoring effect rSO_2_ (%)	100 ± 3	99 ± 4	0.302
Cerebral perfusion: contralateral to surgery			
rSO_2_ at baseline (%)	75 ± 11	74 ± 8	0.759
rSO_2_ at administration (%)	75 ± 10	73 ± 8	0.651
Lowest rSO_2_ (%)	72 ± 10	71 ± 8	0.797
Highest rSO_2_ (%)	77 ± 9	76 ± 9	0.674
Mean rSO_2_ after administration (%)	74 ± 9	73 ± 8	0.762
Restoring effect rSO_2_ (%)	100 ± 3	99 ± 2	0.875

*rSO*_*2*_ cerebral tissue perfusion

*p* value was considered significant < 0.05. Data in mean (standard deviation). Mann–Whitney *U* test was used to calculate the *p* value for nonparametric variables. Restoring effect of rSO_2_ was calculated by dividing ‘mean rSO_2_ after administration’ by ‘rSO_2_ at baseline’ *100

**Table 4:** Absolute change after administration in systemic and cerebral hemodynamics

	Ephedrine (*n *= 15)	Phenylephrine (*n *= 14)	*p* value
ΔMAP (mmHg)			
120 s	23 ± 13	13 ± 14	0.057
Maximum	44 ± 18	27 ± 15	0.011
ΔrSO_2_ ipsilateral (%)			
120 s	− .63 ± .9	0.04 ± 1.9	0.239
Lowest	− 2.3 ± 1.5	− 2.3 ± 1.7	0.978
Highest	3.1 ± 2.5	2.8 ± 1.8	0.739
At highest MAP	− .4 ± 1.9	0.7 ± 2.3	0.657
ΔrSO_2_ contralateral (%)			
120 s	− .9 ± 2.2	− .04 ± 1.3	0.227
Lowest	− 2.5 ± 2.2	− 1.8 ± 1.6	0.327
Highest	2.5 ± 2.1	2.6 ± 1.9	0.813
At highest MAP	.9 ± 2.3	1.1 ± 2.5	0.818
ΔHR (min^−1^)			
120 s	1 ± 4	− 2 ± 6	0.069
Lowest	− 9 ± 7	− 10 ± 9	0.710
Highest	18 ± 13	12 ± 10	0.205
ΔCO (%)			
120 s	− 2 ± 7	− 1 ± 16	0.814
Lowest	− 23 ± 22	− 22 ± 14	0.819
Highest	19 ± 9	20 ± 18	0.874

*CO* cardiac output, *HR* heart rate, *MAP* Mean arterial blood pressure, *rSO*_*2*_ cerebral tissue perfusion

*p* value was considered significant < 0.05. Data in mean (standard deviation). Mann–Whitney *U* test was used to calculate the *p* value for nonparametric variables. Data analyzed from moment of administration to 600 s after administration. Independent samples *t* test

**Table 5:** Relative change after administration in systematic and cerebral hemodynamics

	Ephedrine (*n *= 15)	Phenylephrine (*n *= 14)	*p* value
ΔMAP (mmHg) (%)			
120 s	31 ± 18	19 ± 18	0.081
Maximum	60 ± 27	35 ± 26	0.018
ΔHR (min^−1^) (%)			
120 s	2 ± 12	− 3 ± 9	0.211
Lowest	− 4 ± 11	− 15 ± − 14	0.013
Highest	13 ± 25	22 ± 20	0.318
ΔCO (%)			
120 s	− 2 ± 7.5	− 1 ± 17	0.821
Lowest	− 9 ± 10	− 8 ± 9	0.756
Highest	8 ± 7	5 ± 8	0.379

*CO* cardiac output, *HR* heart rate, *MAP* Mean arterial blood pressure, *rSO*_*2*_ cerebral tissue perfusion

*p* value was considered significant < 0.05. Data in mean (standard deviation). Mann–Whitney *U* test was used to calculate the *p* value for nonparametric variables. Data analyzed from moment of administration to 600 s after administration

## Discussion

Both ephedrine and phenylephrine single-dose administration for correcting intraoperative hypotension during CEA showed to be effective in restoring MAP and are equally effective in preserving rSO_2_ measured by NIRS. Based on the results of rSO_2_ changes and CO, no preference can be expressed in favor of one of the investigated vasopressor agents. In this randomized controlled setting, our results do not confirm the findings of different clinical reports, which described a negative impact of administration of phenylephrine on rSO_2_ [6–8, 19, 20].

Phenylephrine, as a pure α_1_-adrenergic receptor agonist, solely increases peripheral total resistance, devoid of direct effects on cardiac contractility [21]. By stretching the arterial baroreceptors, an increase in MAP results in a baroreflex leading to a decrease in sympathetic activity on the peripheral blood vessels and the heart. This results in bradycardia and a decrease in CO [7, 22]. It is remarkable that in the current study this suspected decrease in HR and CO, as a reflex to the increase in MAP, did not occur after administration of phenylephrine. These findings are confirmed by a recent study, showing that BRS is absent during GA with sevoflurane. This explains the nonappearance a suspected BRS-mediated decrease in HR [23]. No difference was seen in CO after administration between groups. This can be explained by the ambiguous influence of phenylephrine on cerebral hemodynamics. In healthy subjects, phenylephrine administration led to a decrease in rSO_2_ [6]. While SV was not influenced, HR was lowered following phenylephrine administration resulting in a decrease in CO and restraining of cerebral oxygenation [6]. Others endorse the suggestion that administration of phenylephrine increases the arterial pressure, but lowers the rSO_2_, as a consequence of the decrease in CO [7, 8, 24]. Conversely, the impact of a bolus of phenylephrine on CO is also related to the preload dependence of the heart. In preload-dependent patients, no effect of phenylephrine on CO will be expected [24, 25]. Also, the patient population in the present study was vascular compromised, with a high possibility of systemic atherosclerotic vascular disease. This might suggest a different response in CO to phenylephrine in comparison with a healthy patient population [26].

The earlier described decrease in rSO_2_ after administration of phenylephrine, primarily measured in healthy non-cardiovascular patients, is more difficult to explain. We are aware of the fact that phenylephrine does not cross the blood–brain barrier. However, the influence of sympathetic activity on the cerebral blood flow is a matter of ongoing debate. Several studies showed the presence of α-receptors in the smooth muscle layer of the cerebral vessels and possible blood–brain permeability for α-receptors–agonists. In healthy subjects, a change in rSO_2_ determined with NIRS was inversely related to changes in MAP and cerebral blood flow. A reduction in cerebral perfusion has been observed despite an increase in the MAP [27]. This underpins the theory of cerebral vasoconstriction due to an α_1_-effect of phenylephrine after all [11, 12]. Further studies need to be addressed to determine the underlying mechanism behind this theory and to investigate the effect of catecholamines after a period of cerebral ischemia.

Stenosis of the carotid artery, due to its predominantly location in the proximal internal carotid and carotid bifurcation, can reduce the sensibility of the carotid sinus and consequently may impair the BRS [1, 28]. Abnormal HR responses are described to various tests, as the Valsalva maneuver or postural test, in patients with a stenosis of the carotid [29]. Since the patients in this study have severe carotid artery stenosis, there is a high chance an impaired BRS will accompany. We, therefore, hypothesize that patients with carotid stenosis and an impaired BRS respond differently to the administration of phenylephrine: after an increase in MAP, HR is not lowered, and subsequently, CO does not decrease (Fig. 3). Although 43% of the patients in the phenylephrine group used preoperative β-blockers, no significant increase in HR after administration was measured.

Administration of ephedrine causes a release of norepinephrine, hereby stimulating α- and β-adrenergic receptors. This results in an elevation of MAP, HR, and CO [6]. Ephedrine is effective in raising MAP in different scenarios, varying from volatile general intravenous anesthesia to spinal anesthesia. Ephedrine is not associated with a decrease in rSO_2_ after administration [7, 8].

In 2012, Pennekamp et al. [13] described a decrease in rSO_2_ after administration of phenylephrine for treatment of hypotension in patients with carotid artery stenosis undergoing CEA. Unlike these results, no decrease in rSO_2_ was found after administration of phenylephrine in a similar, although larger patient population, in this randomized controlled trial. Although a significant difference in rSO_2_ was found in a small patient population that might suggest a powerful effect of phenylephrine on rSO_2_, this decrease was noticed in only four CEA patients. The small patient population (ephedrine *n *= 7 and phenylephrine *n *= 4) in the study of Pennekamp might have contributed to a distorted view, especially since patients were retrospectively included and not randomized to a treatment arm. This makes considerations of anesthesiologists to administer either phenylephrine or ephedrine for hypotension treatment not transparent. Therefore, we cannot rule out that the decision might be influenced by patient characteristics leading to confounding by indication. Additionally, the retrospective study of Pennekamp was used for power analyses calculation of this randomized controlled trial. Taken the above into consideration, this might have given an underestimation of the sample size and consequently our results [13]. A similar randomized controlled study to ours found a higher restoration of ipsilateral and contralateral rSO_2_ after administration of ephedrine compared to phenylephrine and therefore recommends to prefer ephedrine. However, in our study, no significant difference was found in a decrease in rSO_2_ after administration between both vasopressor agents [30].

### Limitations

In our study, the administrated dose of 50 µg of phenylephrine was less compared to other studies, which used 80–200 µg [6–8]. Nevertheless, the increase in MAP (70 to 87 mmHg after 5 min) in our study is comparable to other studies in which a larger bolus of 80 µg was administrated and reported an increase in MAP from 73 to 86 mmHg or from 72 to 87 mmHg [8, 24].

Secondly, we used sevoflurane or isoflurane for maintenance of anesthesia, in contrast to other studies, who used propofol [6, 8]. Due to the suppressive effect of propofol on the EEG monitoring, both sevoflurane and isoflurane are used regularly during CEA when EEG is monitored intraoperatively to decide whether a shunt is used or not [31]. Sevoflurane impairs CA in high burst-suppression doses and has a vasodilatory effect on the cerebral arteries [32]. It might have blunted the decrease in rSO_2_ after a bolus of phenylephrine. Although a minimum alveolar concentration of 0.5–1 was administered, which is beneath burst-suppression levels, little is known about the influence of sevoflurane on the already impaired CA. It is suggested that normal dose of sevoflurane does not affect an already impaired CA [23]. Of note, within the current study we did not determine individual CA.

Thirdly, we used NIRS as cerebral perfusion monitoring for data analysis. A few reports have demonstrated that the NIRS signal is influenced significantly by extracranial contamination. Oxy-hemoglobin signals were affected by changes in skin blood flow during infusion of norepinephrine, hyperventilation, whole-body heating, injections of ephedrine, and local extracranial hypoxia through a circumferential pneumatic head cuff [33–35]. The clinical implication of this extracranial contamination is uncertain. Several studies consider NIRS as complementing monitoring, to transcranial doppler (TCD) and EEG, for detecting cerebral ischemia [18] Cerebral desaturation as detected by NIRS may be associated with adverse neurological outcomes and prolonged hospital stay [36].

Fourth, all patients in both treatment arms received intravenous low-dose norepinephrine at the moment of administration of the study medication conform standard anesthesiology care during CEA in our hospital. The effect of a single dose of phenylephrine and ephedrine for restoring of MAP after a period of hypotension on rSO_2_ during CEA was determined. The period of hypotension of interest occurred under intravenous administration of norepinephrine. Vasopressor agents in both study arms were additional to intravenous peripheral low-dose norepinephrine to restore MAP. In our belief, this reflects reality concerning the treatment of intraoperative hypotension during CEA. In addition, average given intravenous doses of norepinephrine in both study arms were similar. Therefore, the possible influence of norepinephrine on the results is suspected equally for both study arms. However, a pharmacological effect of both norepinephrine and volatile GA (sevoflurane or isoflurane) in combination with administration of either phenylephrine or ephedrine on the results cannot be excluded. Therefore, the results of this study cannot be directly extrapolated to the awake patient undergoing CEA.

Fifth, gold standard for determination of cerebral blood flow is invasively and time-consuming. A minimally invasive alternative is determination of cerebral blood flow by TCD. Unfortunately, TCD measurements were excluded from analyses due to a lot of missing data and therefore not reliable and useable for this study. Therefore, we were not able to determine the percentage changes in the flow in the middle cerebral artery per patient.

Finally, intraoperative hypotension in this study was defined as a decrease ≥ 20% to the awake baseline MAP. The decrease in MAP at the moment of administration of vasopressor agents was in both groups ≥ 20% compared to awake MAP 1 day before surgery, which is according to protocol [14]. This use of baseline BP was based on a single BP measurement and might not reflect patients’ BP at home due to anxiety-induced stress of being in the hospital facing surgery. However, it is difficult to define a realistic baseline BP measurement which makes a ≥ 20% decrease in MAP intraoperative unclear. The previous study concluded that an optimal reference value or baseline BP for research purposes should be based on a preoperative 24 h measurement at home [37, 38].

## Conclusion

In the present randomized controlled study on intraoperative hypotension control, we did not find a different effect between phenylephrine and ephedrine on frontal cerebral lobe oxygenation in patients undergoing CEA. Both vasopressor agents maintained rSO_2_. Based on our observations, we cannot advise prioritizing the use of one of the agents above the other during CEA.

## Clinical Trial Registration

ClincalTrials.gov, NCT01451294.
